# Mitochondrial Fitness Science Communication: A Qualitative Study

**DOI:** 10.1111/jgs.70019

**Published:** 2025-07-24

**Authors:** Jeffrey T. Boon, Brandon Grubbs, Maulik R. Patel, John Dunavan, Kelly J. Knickerbocker, Cathy A. Maxwell

**Affiliations:** ^1^ Department of Anesthesiology, Division of Anesthesiology Critical Care Medicine Vanderbilt University Medical Center Nashville Tennessee USA; ^2^ Department of Health and Human Performance Middle Tennessee State University Murfreesboro Tennessee USA; ^3^ Department of Biological Sciences Vanderbilt University School of Medicine Nashville Tennessee USA; ^4^ Peer Tree Franklin Tennessee USA; ^5^ Vanderbilt University School of Nursing Nashville Tennessee USA

**Keywords:** geroscience, mitochondrial fitness, older adults, physical activity, science communication

## Abstract

**Background:**

Geroscience explores aging at the cellular level. We developed a lay‐friendly science communication intervention (MitoFit) that addresses healthy aging through mitochondrial fitness. The intervention aims to promote physical activity by educating aging adults about optimizing mitochondrial function to promote healthy aging and prevention of chronic disease. We conducted focus groups to gauge older adults' responses to the video component of the intervention and the effect on their uptake of the intervention.

**Participants and Setting:**

Adults ages 50 and older (*N* = 101; mean age 67.8 years, 75.0% female, 71.7% White, 27.3% Black or African American) participated in one of 16 focus group sessions in community sites in the Nashville, TN, USA area.

**Methods:**

Participants viewed the six MitoFit videos two at a time, pausing after each set of two for focus group discussions of their responses to those videos. Facilitators used a semi‐structured interview guide, and the focus groups were audio recorded. After all focus groups, three study team members analyzed the transcripts using open and axial coding processes. After the discussion of emergent themes from the data, a conceptual model was developed depicting how the science communication approach operated in the sample.

**Results:**

The participants reported overall positive responses to the video quality and content, including recognizing that they were able to understand scientific content about mitochondrial function and its relationship to aging. Participants expressed a sense of having to take action toward physical activity and a sense of hope as a result of the science communication. Our conceptual model suggests that the science communication approach fosters cognitive restructuring, which in turn enhances motivation to engage in physical activity.

**Conclusions:**

The MitoFit scientific communication was well received and should be considered in behavior change strategies that promote physical activity in community‐dwelling older adults.

**Summary**

- Key points
  ○ Mitochondrial fitness, associated with healthy aging and non‐communicable disease prevention, is targeted in MitoFit, a science communication intervention to promote physical activity in older adults by understanding the role of mitochondria in aging.
  ○ Participants who viewed the MitoFit videos found the videos interesting, understandable, and experienced motivation for physical activity as a result of the intervention.
  ○ Science communication approaches promote cognitive restructuring of health beliefs to promote behavior change.
- Why does this paper matter?
  ○ This paper demonstrates the cognitive processes and responses of people viewing science communication (MitoFit videos) about mitochondria in aging and the role of physical activity to impact their mitochondrial fitness. The qualitative data from this study showed that science communication approaches to behavioral health change interventions promote the necessary cognitive restructuring to motivate participants towards change.

## 1 Introduction

Non‐communicable chronic diseases increase with aging and are the leading cause of disability and death [1]. By 2035, over 35% of adults over age 50 will have at least one chronic disease, rising to 47% by 2050 [2]. The chronic disease epidemic is worsening despite billions of dollars in medical expenses [3]. A shift in focus is needed that jolts awareness and attention toward understanding the links between aging, disease, and preventive measures to mitigate these projections. This is the focus of the geroscience movement that explores aging at the cellular level [4, 5]. A recent editorial in the *Journal of the American Geriatrics Society* called for public science communication that garners attention and motivates individuals to act [6]. Evidence shows default reasoning associated with poor decision‐making (e.g., vaccine misperceptions) can also lead to accuracy‐driven reasoning and larger corrective effects through science communication that enables individuals to grasp the implications of their reasoning [7].

In 2019, our team began incorporating science‐based information into interventions to promote healthy aging, reporting strong community interest and improvements in health‐related outcomes [8, 9]. We realized a major gap existed in the public's understanding of aging and development of chronic conditions that pose serious public health problems [10]. To address this gap, we developed a science communication intervention (MitoFit) based on a video series, titled “How to Slow Down Aging Through Mitochondrial Fitness.” Pilot testing for acceptability and feasibility was recently reported with promising results [11]. MitoFit development and testing was internally funded, occurred over 1 year, and was based on the NIH‐stage model [12]. As a science communication approach, MitoFit differs from conventional behavior change approaches by explaining the “why” behind age‐related decline. MitoFit's development was informed by experts in science communication with goals to (a) increase knowledge and understanding about the importance of mitochondria and (b) influence behavior through evidence that choices have consequences [13, 14]. To determine if the goals for the MitoFit videos aligned with our communication approach, the pilot study included focus groups with community dwelling adults aged 50 and older (*N* = 101). Rich and relevant qualitative data emerged from the focus groups, warranting a separate publication. The purpose of this paper is to report the qualitative results from the MitoFit pilot study in which we explored the response to the MitoFit science communication intervention. Here we report the thematic analysis that informed development of a conceptual model based on science communication.

## 2 Methods

### 2.1 Research Design

This qualitative descriptive study was approved by the Vanderbilt University Institutional Review Board (IRB# 230300). Details of the study methods for the full pilot study examining intervention feasibility and acceptability are reported in a prior publication [11]. Using a qualitative descriptive design and established COREQ (Consolidated Criteria for Reporting Qualitative Studies) guidelines [15], we conducted our thematic analysis from deidentified data derived from 101 participants who watched MitoFit videos.

### 2.2 Participants and Recruitment

Participants were recruited via flyers posted in one neighborhood, three senior centers, and one workplace newsletter and email list in the Nashville, TN area. Individuals were eligible to participate if they spoke English, were age 50 or older, and were able to walk independently with or without a walking device. All participants completed written informed consent.

### 2.3 Data Collection

Participants attended a group session, and up to 12 participants were scheduled for each. Sessions were held from May to December 2023. During the sessions, the participants completed a demographic survey which included a question about their exercise habits, “In the past year, have you engaged in a regular exercise routine?”.

In addition to our science communication goals, we were communicating complex scientific content in ways lay people could use the content in everyday life [13]. We postulated the videos would prompt protection motivation by eliciting threat appraisal (perceived vulnerability) and coping appraisal (belief in effectiveness of physical activity), leading to a change in how individuals think about the importance of physical activity that could translate to behavior change [16]. With these goals in mind while developing the content, the videos began with basic information about the form and structure of mitochondria followed by information about how structural features (membranes and DNA) become damaged and how they can be repaired. The videos conclude with practical guidance about how to maintain mitochondrial fitness over time and four short stories from older adults who incorporated mitochondrial fitness into their daily routine. The MitoFit videos were viewed two at a time. Videos 1 and 2 provided an overview of the series and a video on “What are mitochondria?” Videos 3 and 4 focused on “how mitochondria become damaged” and “how to make new and better mitochondria.” Videos 5 and 6 covered “keeping energy requirements in balance” and “putting it all together.” The final video is four short stories on how other adults have improved their mitochondrial fitness.

Following each set of two videos, a session leader led discussions using a semi‐structured interview guide (Figure S1). Focus group questions were based on published goals for communicating science (noted above) [13, 14]. The questions gauged initial reactions, most important take‐aways, suggested changes for making the videos more effective, and the most memorable parts. If participants had questions about the videos, they were asked to wait until the end of the session to prevent information bias. The interview following each set of two videos lasted 10–15 min. The sessions were audio recorded, uploaded to a transcription service (Rev.com), and de‐identified dialogue was transcribed verbatim.

### 2.4 Data Analysis

Transcripts were uploaded to an Excel spreadsheet with a separate sheet for the responses to each set of two videos (Videos 1 and 2, 3 and 4, 5 and 6). Three members of the research team were responsible for coding: the principal investigator, a PhD‐prepared nurse with expertise in geroscience, mitochondrial fitness, aging, and frailty (C.A.M.); a PhD‐prepared nurse with expertise in older adults, pain, and dementia (J.T.B.); and a masters‐level nursing student with prior experience in qualitative coding (K.J.K.). After immersing themselves in the text, each member independently coded the transcripts using qualitative content analysis followed by group reconciliation of coding and updating of the master codebook [17]. We sought to accurately represent the data based on the participants' intended meaning without assumptions. After completing coding for all focus groups, we discussed the emergence of themes from individual codes and relationships among the themes [18]. We subsequently developed a conceptual model depicting how a change in thinking (cognitive restructuring [CR]) occurs through a science communication approach.

## 3 Results

One hundred one individuals participated in 16 group sessions. The participants had a mean age of 67.8 years, were predominantly female (75.0%), and two‐thirds had some college or a degree (52.0%). Among the participants, 27.3% identified as Black, while 71.7% identified as White. Sixty‐nine (71.1%) of 97 participants identified that they had engaged in regular exercise in the past year. A summary of sample characteristics is shown in Table 1.

**TABLE 1:** Participant characteristics.

Characteristic	*N*	Mean (SD), min, max
Age (years)	101	67.8 (8.9), 50, 87
Age Group		n (%)
50–59		18 (17.8)
60–69		40 (39.6)
70–79		34 (33.7)
80+		9 (8.9)
Race	99	
Asian		1 (1.0)
Black/African American		27 (27.3)
White/European American		71 (71.7)
Gender	100	
Female		75 (75.0)
Male		25 (25.0)
Marital/Partnered Status	101	
Divorced/Single		34 (33.7)
Married/Partnered		48 (47.5)
Widowed		19 (18.8)
Education Level	100	
H.S./GED		14 (14.0)
Some college/Associate		33 (33.0)
Bachelor's degree		29 (29.0)
Masters degree		13 (13.0)
Doctoral degree		11 (11.0)
Employment status	101	
Full time		26 (25.7)
Part time		9 (8.9)
Unemployed		4 (4.0)
Retired		62 (61.4)

Over 1000 quotes were analyzed through an inductive and iterative process. The results of coding the focus group interviews fell into two large categories: (a) comments about production quality and presentation, and (b) comments reflecting the participants' responses to the video content (science communication) and messaging about mitochondrial fitness and health. Exemplar quotes are shown in Tables 2 and 3 and embedded within the narrative. Full counts of the code frequencies are shown in Tables S1 and S2.

**TABLE 2:** Exemplar quotations for codes describing video production in the transcribed interviews.

Code	Exemplar quotations
Good visuals	“And the amazing part for me was the working together of this system. The working together that we fail to remember that we don't… not just one thing goes, everything has to work together in order for us to really benefit. And I could see it. I could see it in the graphics. I could. It was very visual to me. I could see the thinness of the membrane. I could see… I believe that for me that really, I have a picture of it now.” “Well, I'd never seen anything that showed what it really looked like inside [a mitochondrion]. I've read a lot, but I've never really had that so clearly shown.”
Appreciate diversity	“You showed different stretching and walking and there's other stuff too. Lifting…doing weights. I like that.”
Engaging	“When I saw the ATP [adenosine triphosphate] come up, I thought, ‘Uh‐oh. This might go too far,’ but actually it didn't…. I get it. I thought the production was very nice and very engaging.”
Understandable	“I think it's very basic, the way it was explained. That it is not a technical jargon that you can't understand. It's like, man, I'm excited about this because there is something we can do and having the foundation of what is actually happening to the cells.”
Informative	“It was very informative, and it explained why sometimes you don't have energy to do something.”
Relatable	“I love the personal stories because that makes it real, and it makes it achievable. It's not just something out there.”
Interesting	“I think it certainly piqued my interest to listen to the next ones [videos].”
Makes sense	“I don't have a big background in biological sciences at all, and it made sense to me.”
Too much detail	“The second video started to get a little bit more technical” “I was trying to think, ‘Oh, I've got to absorb all this, but it's coming too fast. And so, if I could have just been reminded the whole purpose…was just why it's important, not to try to understand everything yet.”
Change wording	“You said, ‘You have millions of [mitochondria] in your body,’ and that's sort of an ‘ick’ factor to it. I would have said, ‘They're perfectly natural. They're normal. They're part of your system.’ Because, at first, it's like, ‘Do you mean like bacteria? Do I have an infection? What are you talking about?’”
Needs tailoring	“[For some individuals, walking] outside in their neighborhood might not be the safest thing to do.” “Options and the ability to choose something that you would do [are important for MitoFit].” “You can start now and that it can be personalized. Can be a personalized plan for you, and you can fit it into your lifestyle. So what's right for me may not be right for [another participant], but you can make it fit your lifestyle.”
Need more information	“I'm still curious about food. Because it really didn't talk about food at all.”
Multifactorial	“it's not just one thing, exercise. It's a multitude of things that keeps the body healthy as we age”

**TABLE 3:** Exemplar quotations for codes describing responses to mitochondrial fitness and health content in the transcribed interviews.

Code	Exemplar quotations
Impactful	“It really grabbed me.” “[It was] impactful to understand the charge of the energy and seeing the battery go down and charge.”
Knowing why	“I think what I learned first is that when you go to the doctor and he tells you [that] you need to exercise more, you need to eat better, he never tells you why. And this is telling you why.”
Motivating	“[The videos make me] want to exercise, which I don't want to do.”
Real testimonies	“[The testimonies] bring it home and make it more real and kind of a personal touch.” “[The testimonies] brought everything together. This is real life. This is real people being impacted.”
Making a connection	“I'm connecting the how for me because sometimes that could be the missing piece” “We always think about the external….this is hurting, this is bruised, or whatever…. Everything's always starting from the inside out….We don't always think about what's going on the inside….We never think about cellular wise.”
“This is biology”	“[My excitement came from] understanding what is actually happening to the cells.” “it's not a gimmick—this is biology.”
Energetics	“Your energy, how much oxygen you can get into, and that type of thing.”
Battery analogy	“I didn't realize each cell had its own little series of batteries…. And it looked like there were several of them inside the one cell model that you showed there. I think everybody knows that the more you exercise, the more energy you're going to have, but I don't think anybody knew why.”
Cause for concern	“[It was] a little bit scary for a minute….because you're like ‘What have I done to myself over time? Thank God I don't smoke.’ That kind of thing.”
Unaware	“I always think of exercising as good for your muscles, and your heart is a muscle. But this is saying that also all of your organs. Never thought about it that way.”
Have to do it	“If you want to increase your energy level, you've got to apply yourself to it. You can't just want it to be.” “As we age, we should understand that we can't sit and just do nothing because then that starts the slowing down process even faster. So if we get a better understanding that we keep moving, then we don't need too many batteries so fast.”
Finding what I like	“The answer is not just going to the gym and running on a treadmill, or just doing this, but find something that I enjoy doing and make that a habit. Be consistent.”
Making a plan	“You got to get a plan for yourself of physical activity of some sort. It'll be different for everybody, but we have a personal plan.”
Hope	“The one word I would come at is hope. We talked about how things get damaged, and now they gave us some solutions to it.”
Not too late	“I am very grateful that I must have done something right, that it's never too late. That right now I'm getting the message. You're still not too late. You are here, and you're listening that you've got a chance of actually doing some changing, including at my age.”
Desire to share	“I would like that for people who are coming to be my age to begin to know that a little bit sooner” (regarding the ability to make changes).
Sense of agency	“[I like] that you have some control over the health of your mitochondria. I like that.”
Doing it for me	“I feel very fortunate to be getting this information at this point. That I can hopefully make a difference for my body.”

### 3.1 Video Production Comments

Responses related to video production resulted in 45 codes from which we identified three themes: production quality, positive comments, and comments for improvement.

#### 3.1.1 Video Production Quality (5 Codes, 45 Quotes)

The participants commented on the visual appeal of the MitoFit videos, especially for visualizing cellular processes. Many discussed how the visualization of mitochondria helped them to understand the interaction of mitochondria with other processes in the body. The participants also noted there was value in seeing people doing different types of physical activity.

The participants stated the delivery of the videos was engaging. They noted the appealing nature of the videos, allaying their fears that learning about mitochondria might be too complex. Most participants thought the pacing of the videos was good, and they appreciated seeing options and variety.

#### 3.1.2 Positive Comments (13 Codes, 136 Quotes)

Participants provided positive feedback about the videos themselves with a high frequency of codes for *understandable* (38), *good* (29), *informative* (25), *relatable* (24), and *interesting* (20). Despite the potential for complexity of biological information about mitochondria, most participants were able to understand the videos and found them engaging.

Relatability of the videos was key to the positive reaction. The final video shows stories of individuals who experienced adverse health events and have begun working on their mitochondrial fitness. These videos were noted to increase the relatability of the entire video series' content. An additional appeal of the MitoFit videos is that while they were relatable, they were also interesting; participants found the information something they wanted to continue to watch. Other responses included the way the material was presented. It made sense to the participants even without significant prior scientific knowledge. Most participants also liked the length of the videos and found the suggestions actionable and helpful.

#### 3.1.3 Comments for Improvement (28 Codes, 136 Quotes)

The research team sought feedback to improve the MitoFit videos. The codes with the most frequent quotes were *confusing* (21 quotes) and *needs tailoring* (20 quotes). One participant stated “[the term] escort came and ATP. Those were two words I was like, ‘Wait a minute, what does that mean?’” Another said, “if someone has never heard any of the terms before, then it may be hard to follow, especially if they don't jive the lead‐in and the narration, might need a little bit more explanation.” Codes for *too much detail* (13 quotes) and *change wording* (14 quotes) were primarily in response to the second video in which mitochondrial structure and functions were described.

Some participants commented that the *pacing was too fast* (8 quotes), a video was *too long* (2 quotes), or that *context was needed* (6 quotes) such as adaptations for disabilities. Some participants responded that the testimonies of individuals describing their health and lifestyle changes in the context of mitochondrial health felt too scripted.

Other participants commented on areas of health such as nutrition that were not included in the videos but interested them. This goes along with another impression expressed by participants that even though the focus of MitoFit is physical activity, there are many aspects of health promotion.

Participants also suggested there may be a value to support materials for viewers because of the volume of new information. While some participants noted some preparatory information in the early videos (e.g., structure and function of mitochondria) did not seem relevant, they also stated they may not yet see its relevance. This code did not recur in responses to later videos.

### 3.2 Responses to Mitochondrial Fitness and Health Content

Responses related to information and health content in the videos produced 45 codes from which we derived 11 themes. In the section below, a brief description of each theme is provided with example quotes.

#### 3.2.1 Themes

The theme *proactive reactions* reflected positive, impactful perceptions about the content. The most common code (59 codes) was *impactful* (realization of the importance of the information). One participant stated, “I keep thinking you have to know what this is. If you don't know, how can you do something about it?” The code *motivating* (49 quotes) reflected that the videos served as a stimulus to engage in physical activity, such as “But watching that, it incites you. It lets you know that ‘Hey, look, just because you're older, you need to do something.” Other codes within proactive reactions included *real testimonies* (20), *knowing why* (19), *making a connection* (13), *fascinating* (11), *leaves you wanting more* (8), *enjoyable* (5), and *amazement* (3).

The theme *this is biology* (43 quotes) reflected realizations of the scientific importance and application of information to the human body. For example, from Videos 3 and 4, a participated stated, “The mitochondria is a good introduction to awaken us that we have to do something to stay healthy and live a productive life, because doing nothing guarantees us that won't happen.” Another stated, “…the reality that it's scientific makes it less of a moral judgment and more like ‘this is how it is, this is your machine.’” Minor codes within this theme included *not a gimmick* (1) and *removal of moral judgment* (1).

*Energetics* reflected recognition that mitochondrial fitness involved one's personal experience of energy or fatigue. One participant said, “When you're sitting around and not doing anything, you get tired. Why is that? You [think you] should be conserving energy, but actually it takes energy to make energy. So then, when you get out and exercise, you start to get that energy back.” Also under this theme, the code *steady state* (3) was observed in the statement that “for me it was that keeping the steady supply and demand, keeping it steady and not just saying this week I'm taking it easy but just keep it.”

The theme *cause for concern* (35 quotes, 8 codes) was defined as the realization that failure to engage in regular physical activity could have detrimental effects. Within this theme, 31 of 35 quotes came from Videos 3 and 4. One participant said, “because you're like, ‘What have I done to myself over time?’” Another stated, “I think out of everything I've seen, the thing that scares me the most is the DNA breakdown.” The code *free radicals* (27) reflected sentiments of alarm such as “What do you do about the free radicals? She doesn't even know what it is. I knew they were harmful. I knew they damaged cells, but I didn't realize that your own body generates them. And how do I get rid of them?”

Thirty‐six quotes reflected the theme *unaware*, defined as acknowledgment of not knowing about the information in the videos. Example quotes include, “Never thought about it that way” and “but I like that it lists out what causes the damage and so it gives you a pause in how to avoid those or what to think about going forward. And then I also liked having the quantity of the minutes [that one should exercise] outlined there.” Other codes within this theme included *new knowledge* (29) and *mitochondria/battery analogy* (2).

The theme *leads to more questions* (48 quotes) was defined as expressions of needing to know more. Additional codes were *sparks interest* (2) and *tells why the need to know* (2). Content on free radicals led to some of the quotes within this theme such as “throughout the whole thing I'm thinking ‘Okay, what are free radicals?’”

*Have to do it* was the theme with the most quotes (264 total for theme, 94 quote for the code have to do it), defined as realizing physical activity is necessary for mitochondrial fitness and health. Sixteen codes were identified under this theme, reflecting a broader effect of the videos on the participants' thought processes. One participant stated, “It really showed me the need of how you really need to do some type of exercise. Period. That's what I got out of it.” Another said, “You can't give up. You got to keep going. It is a reminder how important it is,” and another expressed, “Yeah, and I thought that the takeaway was kind of, you need to be intentional about it, even though you can get your 10,000 steps in and not know it. But to get the cardiac increased heart rate, you probably have to be intentional about it and the strength training.” Codes within this theme included *consistency* (37), *exercise* (37), *finding what works* (15), *finding what I like* (21), *commitment to health* (8), *start early* (9), *making a plan* (17), *finding balance* (8), *staying aware* (3), *need accountability* (3), *building momentum* (3), *keep it simple* (3), *nutrition* (3), *cumulative effect* (1), and *intensity* (1). The number of quotes within this theme grew as participants viewed the videos with 22 from Videos 1 and 2, 87 from Videos 3 and 4, and 155 from Videos 5 and 6.

The theme [and code (64)] of *hope* (74 quotes, 2 codes) reflected perceptions that physical activity could be beneficial at any age. A participant stated, “I think that this is telling me and encouraging me with the idea that ‘Yes, it is going to make a difference. It is worth the effort’. So, I see that as encouraging in that way.” Another stated, “Well, the most important one I made note of is you can start at any age.” The code *not too late* (10) was also under this theme.

The theme [and code (13)] of *individualism* is defined as reflections of individual differences between self and others. One participant stated, “I think, too, the options. Not everybody is the same. Nobody's the same, and so there are certain limitations that, I mean, everybody has that either they never asked for or they created in their own body.” Similarly, the theme *sense of agency* reflected expressions of self‐efficacy and an ability to accomplish. A participant said, “I do a lot of things by myself, but I do it for me because I have this condition, and if I don't do it, I will just get stiff and won't be able to take care of myself.” The code *doing it for me* (5) was also included in this theme.

A final theme [and code (9)], *desire to share*, reflected wanting others to know about the Mitofit information. One person expressed, “Greatly appreciate it that this [the videos] is being done for those of us who need it and for those who don't know they need it yet.” Another said, “Grab a person who doesn't exercise and pass on the information to the next person.” The code *everybody should know* (9) also reflected this theme.

## 4 Discussion

Our results revealed overall positive responses to the MitoFit videos.

Most conveyed favorable impressions of the quality of the videos. The comments for improvement were also helpful and will be used to make adaptations to the MitoFit intervention.

Consistent with our goals for the videos, the participants found the presentation of basic science information on mitochondria understandable, interesting, and useful. One key aspect participants identified as helpful in this scientific communication intervention was the analogy comparing mitochondria to batteries. Metaphor and analogy have been recognized as key components of how science is often communicated and as having potential pedagogical benefit [19, 20].

**FIGURE 1:**
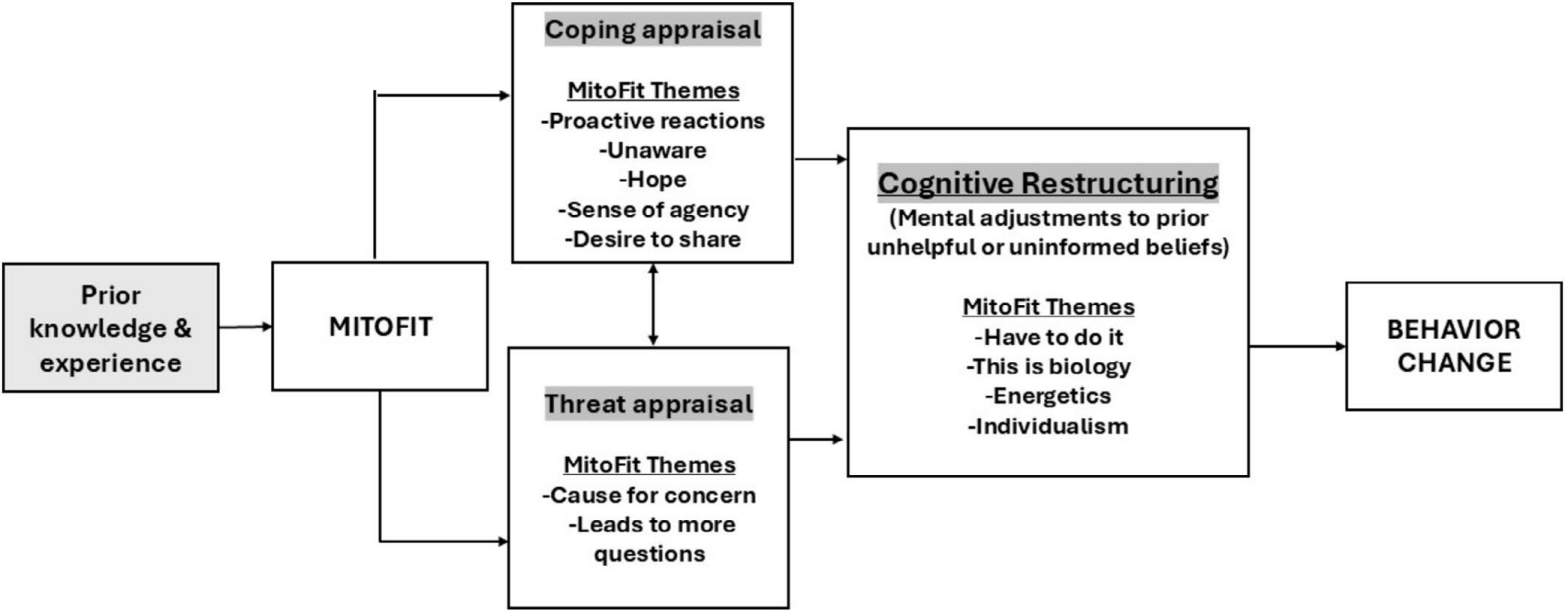
Conceptual model of MitoFit themes with constructs of protection motivation theory and cognitive restructuring, leading to behavior change.

We utilized Protection Motivation Theory (PMT) as a foundation for our work, and the results from this study are consistent with that as our premise. A conceptual model (Figure 1) illustrates how individuals watch the MitoFit videos with differences in prior knowledge and experience. After viewing the videos, five identified themes are consistent with coping appraisal (belief in the effectiveness of the recommendations), and two themes are consistent with threat appraisal (perceived vulnerability). Four themes—including the code with the most quotes (*have to do it*)—reflected the process of CR, defined as a change in maladaptive thinking and replacing it with constructive and practical alternatives [21]. CR is a core element of cognitive behavioral therapy, facilitating a more balanced perspective and considering a broader range of possibilities based on evidence [21]. The CR process was reflected in such quotes as “I need to make sure I'm getting up and I'm doing something every single day,” “you can't afford not to [exercise],” and “you can actually recharge the battery that way and take care of damaged mitochondria.”

Themes, codes, and quotes derived from the participants who watched the MitoFit videos reflected the constructs shown in our conceptual model. As reported in our prior publication, the subsample of participants who completed the MitoFit intervention demonstrated increased physical activity (behavior change) [11].

From a research perspective, communicating science effectively to the public is understudied. Experts indicate the field of science communication will advance through (a) partnership between researchers and clinicians; (b) opportunities and mechanisms of collaboration for developing unified theories, concepts, and definitions; (c) greater recruitment of scientists; and (d) mechanisms for the rapid review and funding of science communication research [13]. Our study makes a contribution to this advancement. We propose that a science communication approach like MitoFit to achieving lifestyle change is more successful than traditional approaches because it goes beyond recommendations by explaining “why” the recommendations work in a way the public can understand. We intend to test this hypothesis in a future study.

### 4.1 Strengths and Limitations

The sample had a good age distribution as well as African American representation. However, other racial/ethnic identities were not well represented. Likewise, the participants were English‐speaking Americans from the southern United States and had higher educational attainment. Due to audio quality, transcriptionists were unable to provide an identifier for every participant, limiting our ability to analyze our results by subgroups (e.g., those who exercised, race, gender, educational level). Such analysis could have provided valuable insights about how science communication is perceived. Finally, while we identified the process of CR occurring immediately after the participants viewed the videos, we did not have significant follow‐up except with a subsample of the pilot group for the MitoFit physical activity intervention [11]. It is unclear how long or well the CR endures or what may be needed to sustain it.

Moving forward and based on our results, we intend to develop additional MitoFit content based on recommendations from the study with continued testing through research approaches. Of note, the MitoFit videos are co‐owned by the PI and Vanderbilt University and will also be available (summer 2025) through the learning platform HealthStream.

## 5 Conclusion

Science communication and targeting of mitochondrial fitness has the potential to achieve behavior change among older adults. MitoFit presents information about mitochondrial fitness to promote physical activity to mitigate the effects of aging and onset or worsening of chronic disease. Our results suggest older adults who watch the MitoFit videos undergo a process of CR, increasing their intention to engage in physical activity. Communicating scientific information to lay people as part of psychoeducational interventions to promote health behavior change represents a novel model for intervention development.

## Author Contributions

Study concept and design: C.A.M., M.R.P., J.D. Acquisition of subjects and/or data: C.A.M., J.T.B. Analysis and interpretation of data: C.A.M., J.T.B., K.J.K. Preparation of manuscript: C.A.M, J.T.B. Approval and revision of final manuscript: all authors.

## Disclosure

The sponsor did not influence this study's design, methods, subject recruitment, data collection, analysis, or preparation of this paper.

## Conflicts of Interest

The authors declare no conflicts of interest.

## Supporting information


**Figure S1.** Semi‐structured interview guide (page 2).
**Table S1**. Frequency of codes represented across MitoFit videos: Video production quality and presentation (pages 3–4).
**Table S2**. Frequency of codes represented across MitoFit videos: Participants’ responses to content about mitochondrial fitness and health (pages 5–6).
