# Supplementary material for: Mitochondrial Fitness Science Communication: A Qualitative Study
Source: J Am Geriatr Soc. 2025 Jul 24;73(10):3065–73. doi: 10.1111/jgs.70019 (PMC12554835; doi:10.1111/jgs.70019)
Supplement: Supplementary file 1 — Figure S1. Semi‐structured interview guide (page 2). Table S1. Frequency of codes represented across MitoFit videos: Video production quality and presentation (pages 3–4). Table S2. Frequency of codes represented across MitoFit videos: Participants’ responses to content about mitochondrial fitness and health (pages 5–6). [file JGS-73-3065-s001.pdf]

Supplementary Materials for “Mitochondrial Fitness Science Communication: A Qualitative Study”

Contents

Supplementary Figure S1. Semi-structured Interview Guide (page 2)

Table S1. Frequency of codes represented across MitoFit videos: Video production quality and presentation. (pages 3-4)

Table S2. Frequency of codes represented across MitoFit videos: Participants’ responses to content about mitochondrial fitness and health. (pages 5-6)

Supplementary Figure S1. Semi-structured Interview Guide

Semi-structured Interview Guide:

1. What is your initial reaction to the videos using one word?

2. What do you think is the most important takeaway from the videos?
   Probes:
   - What new information did you learn?
   - How did the videos help you better understand mitochondrial fitness?

3. What changes, if any would you suggest to make the video more effective?

4. What was the most memorable part of the videos for you?
   Probes:
   - How did the videos help to improve your knowledge and understanding of mitochondrial fitness?
   - How could you use the information from the videos to help you in your day-to-day life?

Table S1. Frequency of codes represented across MitoFit videos: Video production quality and presentation.

Category	Codes	Video 1-2	Videos 3-4	Videos 5-6	Total
**Video production quality**	Good visuals	16	8	4	28
	Appreciate diversity	3	2	4	9
	Good pace	2	4	0	6
	Engaging	1	0	0	1
	Options and variety	0	1	0	1
**Positive comments**	Understandable	20	12	6	38
	Informative	13	7	5	25
	Relatable	4	2	18	24
	Interesting	18	2	0	20
	Good	15	11	3	29
	Simple	9	1	1	11
	Helpful	3	1	4	8
	Makes sense	6	0	0	6
	Liked length	2	2	1	5
	Accessible	3	0	1	4
	Actionable	2	0	0	2
	Nice	1	0	0	1
	Affirming	0	0	1	1
**Comments for Improvement**	Confusing	11	7	3	21
	Too much detail	3	6	4	13
	Change wording	5	5	4	14
	Pace too fast	1	2	5	8
	Need context	1	1	4	6
	Scripted testimonies	0	0	5	5
	Address disabilities	0	0	1	1
	Too long	2	0	0	2
	Need bigger picture	3	0	0	3
	Need more visuals	1	0	1	2
	Distraction	0	0	2	2

	Need more detail	0	6	0	6
	Make actors relatable	0	1	0	1
	Irrelevant	0	0	1	1
	Make it more fun	0	0	1	1
	Redundancy	0	0	1	1
	Too late for me	0	0	1	1
	Space out viewing	0	0	1	1
	Needs tailoring	5	1	14	20
	Need more information (motivation)	0	0	10	10
	A lot to absorb	0	6	1	7
	Multi-factorial	5	0	0	5
	Support material	0	1	3	4
	Consider audience	3	0	0	3
	Lots of information	2	0	0	2
	Not relevant (yet)	5	0	0	5
	Provides context	0	1	0	1
	Add clarity	0	0	6	6

Table S2. Frequency of codes represented across MitoFit videos: Participants' responses to content about mitochondrial fitness and health.

**Theme**	**Codes**	**Video 1-2**	**Videos 3-4**	**Videos 5-6**	**Total**
**Proactive Reactions**	Impactful (wanting)	13	13	33	59
	Motivating (wanting)	8	11	30	49
	Real testimonies (wanting)	0	0	20	20
	Knowing why (non-wanting)	3	9	7	19
	Making a connection (non-wanting)	5	7	1	13
	Fascinating (wanting)	6	2	3	11
	Enjoyable (wanting)	1	0	4	5
	Amazement(wanting)	3	0	0	3
	Leaves you wanting more(wanting)	0	3	5	8
**"This is biology"**	"This is biology" (non-wanting)	27	15	1	43
	Not a gimmick (non-wanting)	1	0	0	1
	Removes moral judgment (non-wanting)	0	0	1	1
**Energetics**	Energetics (non-wanting)	15	6	8	29
	Steady state (non-wanting)	0	0	3	3
**Cause for concern**	Cause for concern (relief)	0	8	0	8
	Free radicals (relief)	0	23	4	27
**Unaware**	Unaware (non-wanting)	14	11	7	32
	New knowledge (non-wanting)	9	12	8	29
	Mitochondria (non-wanting)	2	0	0	2
**Leads to more questions**	Leads to more questions (wanting)	12	19	13	44
	Sparks interest (wanting)	2	0	0	2
	Tells WHY need to know (non-wanting)	2	0	0	2

**Have to do it**	Have to do it (non-wanting)	19	29	46	94
	Consistency (non-wanting)	0	12	25	37
	Exercise (non-wanting)	1	18	19	37
	Finding what works (non-wanting)	0	0	15	15
	Finding what I like (non-wanting)	0	8	13	21
	Commitment to health (wanting)	0	3	5	8
	Start early (wanting)	0	4	5	9
	Making a plan (wanting)	0	10	7	17
	Finding balance (non-wanting)	0	0	8	8
	Staying aware (non-wanting)	0	1	2	3
	Need accountability (relief?)	0	1	2	3
	Building momentum (non-wanting)	0	0	3	3
	Keep it simple (non-wanting)	0	0	3	3
	Nutrition (?)	2	1	0	3
	Cumulative effect (non-wanting)	0	0	1	1
	Intensity (?)	0	0	1	1
**Hope**	Hope (wanting)	9	34	21	64
	Not too late (relief)	0	3	7	10
**Individualism**	Individualism (non-wanting)	5	4	4	13
	Doing it for me (non-wanting)	0	2	3	5
**Sense of agency**	Sense of agency (non-wanting)	10	16	0	26
**Desire to share**	Desire to share (?)	1	2	6	9
	Everybody should know (?)	1	5	3	9
